# Point-of-care ultrasound in nutrition assessment and enteral nutrition management of critically ill children: a scoping review

**DOI:** 10.3389/fnut.2026.1849240

**Published:** 2026-06-22

**Authors:** Yan Li, Min-Jie Ju, Cong-Hui Fu, Xiao-Ya Yang, Ji Liu, Ting-Ting Xu

**Affiliations:** 1 Department of Critical Care Medicine, Shanghai Children’s Hospital, Shanghai Children’s Hospital, School of Medicine, Shanghai Jiao Tong University, Shanghai, China; 2 Department of Nursing, Shanghai Children’s Hospital, Shanghai Children’s Hospital, School of Medicine, Shanghai Jiao Tong University, Shanghai, China

**Keywords:** critical illness, enteral nutrition, nutrition, pediatrics, ultrasonography

## Abstract

**Background and aims:**

Enteral nutrition is essential for improving outcomes in critically ill children, but its implementation is challenged by difficulties in nutrition assessment, feeding tube placement, and gastrointestinal function monitoring. Point-of-care ultrasound (POCUS) has emerged as a potential bedside tool to support these processes. However, its applications in critically ill children remain heterogeneous and lack standardization. This scoping review aimed to map the current applications and characteristics of POCUS in nutrition assessment and enteral nutrition management in this population.

**Methods:**

This scoping review was conducted in accordance with the Joanna Briggs Institute methodology and reported following the PRISMA-ScR guidelines. Seven English databases and four Chinese databases were systematically searched since establishment to 30 November 2025. Articles reported on the POCUS in nutrition assessment and enteral nutrition management of critically ill children were eligible for inclusion and data extraction. Data were extracted and synthesized descriptively. The protocol was prospectively registered on Open Science Framework^1^ on 12 December 2025.

**Results:**

The initial search identified 432 studies, and 32 were included in this review. This scoping review demonstrates a recent and strong focus on this field, with 65.6% (*n* = 21) of the included studies published within the last 5 years. The majority (*n* = 16) were prospective cohort studies. POCUS was applied across three primary functions in critically ill children’s nutrition management: muscle mass assessment (*n* = 20), facilitation of feeding tube insertion and positioning (*n* = 6), and measurement of gastric residual volume and motility (*n* = 6). Substantial heterogeneity was identified in ultrasound protocols, anatomical landmarks, and measurement indicators across studies.

**Conclusion:**

Current evidence suggests that POCUS has diverse bedside applications in nutrition assessment and enteral nutrition management in critically ill children, particularly for muscle mass evaluation, feeding tube management, and gastrointestinal function assessment. Despite growing research interest in this field, evidence remains methodologically inconsistent, limiting the comparability and clinical translation of findings. Future research should prioritize the development of standardized protocols and training frameworks to support the integration of POCUS into pediatric critical care nutrition practice.

**Systematic review registration:**

https://osf.io/k4acf/, osf.io/k4acf.

## 1 Introduction

Enteral nutrition management represents a systematic process of assessment, delivery, and monitoring designed to preserve gut function and meet metabolic demands (1). In critically ill children, it is an integral component as its adequacy is intimately linked to clinical outcomes (1, 2). Critically ill children often experience increased nutrient demands from metabolic stress and inflammation (3), yet often suffer from anorexia, fluid restriction, procedural fasting, and feeding intolerance, leading to cumulative macronutrient deficits and increased undernutrition risk (3, 4). Studies reported that 22.1 to 51.2% of critically ill children are affected by undernutrition (5–7), which weakens the body’s immune defenses and impairs respiratory muscle function (1, 8). Consequently, it is associated with prolonged hospitalization, higher morbidity and mortality, extended mechanical ventilator, and increased risk of organ dysfunction, intensive care unit-acquired weakness (ICU-AW), and hospital-acquired infections (1, 8–10). To improve the nutritional status of critically ill children, enteral nutrition is recommended as the preferred feeding strategy, given its benefits in preserving gastrointestinal mucosal integrity, promoting immune function, and maintaining gut microbiota stability (11). Early enteral nutrition significantly reduces mortality in the pediatric intensive care unit (PICU) and 90-day hospital mortality, along with the duration of mechanical ventilation and PICU length of stay (12). Despite the established benefits of enteral nutrition, its effective delivery to critically ill children face several barriers, particularly concerning the accuracy of nutritional assessment, radiation exposure, and the objective evaluation of gastric emptying (13).

These barriers primarily manifest in several key aspects of clinical practice. Regular and precise assessment of nutrition status plays a key role in identifying nutritional risk and monitoring the efficacy of nutrition therapy (14). Patient weight, while the most commonly used indicator, is often technically difficult to measure and can be influenced by volume status during acute illness (14). Inaccurate assessment of nutritional status directly compromises the implementation of nutrition plans. During implementation, confirming feeding tube placement often requires X-ray examination, and repeated tube placement may increase the child’s risk of radiation exposure (15). Additionally, the assessment of gastric emptying, reflected in feeding tolerance, is a primary determinant for initiating and advancing enteral nutrition (12). A recommendation from the Society of Critical Care Medicine and American Society for Parenteral and Enteral Nutrition calls for the inclusion of bedside support to guide the detection and management of feeding intolerance (1). Gastric residual volume (GRV) is the most common indicator for assessing feeding intolerance and is usually measured via syringe aspiration, while its accuracy may be affected by tube position, patient posture, and aspiration technique (16, 17). Inaccurate assessment of GRV may lead to unnecessary interruptions in feeding, consequently compromising the delivery of enteral nutrition (18). To overcome these barriers, there is a clear need for accurate, non-invasive methods to continuously assess nutritional status, feeding tube position, and feeding tolerance in critically ill children. Point-of-care (POCUS) offers a promising solution, providing a real-time, visualization-based approach to address these clinical challenges (19).

Integrating POCUS into the enteral nutrition strategy holds potential value across the nutrition assessment and enteral nutrition management (20, 21). POCUS can assess muscles such as the quadriceps to accurately evaluate muscle mass, helping to determine nutritional reserves and detect muscle loss early in critically ill children (19). Serial POCUS measurements during hospitalization can serve as an objective indicator for monitoring the adequacy of energy and protein intake, providing a reference for the adjustment of nutritional strategies (22). During the delivery of enteral nutrition, POCUS is also employed for feeding tube localization and the assessment of gastrointestinal function (21). Under POCUS guidance, a standard four-step procedure for postpyloric feeding tube insertion was followed, which resulted in a significantly high first-pass success rate in critically ill children (23). POCUS also serves as an accurate method for verifying nasogastric tube position and has the potential to decrease reliance on confirmatory X-ray, thereby reducing ionizing radiation exposure (21). In pediatric surgical patients, POCUS has been recommended by the European Society of Anesthesiology and Intensive Care for assessing GRV (24). By combining POCUS measurement of the gastric antrum cross-sectional area (CSA) with a qualitative assessment of content in the antrum, GRV can be estimated with greater accuracy than with conventional syringe aspiration (25). Meanwhile, POCUS is typically performed without sedation due to its rapid and painless imaging process, a critical advantage for critically ill children (19). Its capacity for accurate and rapid imaging also makes it well-suited for ethical research settings and for evaluating non-communicative patients, such as those who are unconscious or very young (26). In 2020, the European Society of Paediatric and Neonatal Intensive Care confirmed that POCUS can enhance patient safety and provider performance (27–29).

POCUS extends clinical assessment and enhances procedural safety (30, 31). However, research in this field remains in its early stages, with evidence being relatively heterogeneous and predominantly derived from single-center explorations. Another major challenge is the lack of standardized implementation protocols, including the timing of POCUS assessments, patient positioning during GRV measurement, and the selection of target skeletal muscle groups (32), as well as the optimal measurement sites, specific metrics, and the degree of transducer compression. Currently, there is a lack of reviews summarizing the scope of application, specific methodologies, efficacy, and challenges of POCUS in the enteral nutrition management of critically ill children. Therefore, this scoping review aims to map the existing evidence, identify knowledge gaps, and suggest directions for future research.

## 2 Methods

### 2.1 Protocol registration

We conducted this scoping review according to the PRISMA extension for scoping reviews (PRISMA-ScR) (33). The protocol was prospectively registered on Open Science Framework on 12 December 2025.

### 2.2 Eligibility criteria

We included full-text, peer-reviewed articles published in English and Chinese through November 2025. Studies were included if the following criteria were met: (i) participants aged 29 days to 18 years; (ii) reporting on the application of POCUS in enteral nutrition management; (iii) admitted to PICU. Study design encompassed review, cohort study, cross-section study, diagnostic test accuracy study, experimental research, and case report. We excluded qualitative studies, opinion pieces and conference abstracts (e.g., editorials). In this review, POCUS is defined as bedside ultrasonographic imaging that is acquired, interpreted, and integrated into care immediately by the treating clinician (30).

### 2.3 Search strategy

A systematic and comprehensive literature search was conducted on 19 December 2025 in seven English databases and four Chinese databases: MEDLINE, EMBASE, PubMed, Web of Science, Cochrane Central Register of Controlled Trials, Joanna Briggs Institute Evidence-Based Practice (JBI EBP), CINAHL, China National Knowledge Infrastructure (CNKI), Chinese Science and Technology Periodical Database (VIP), WanFang Data, and Chinese Biomedical Literature Database (Sinomed). Reference lists of included studies were reviewed to identify further studies meeting the eligibility criteria. Initially, a preliminary search was performed in PubMed and CNKI databases, combining MeSH terms and keywords to pilot and assess the suitability of the search strategy, keywords, and databases. The initial PubMed search query consisted of: (child OR infant OR pediatrics) AND (critical care nursing OR intensive care units) AND (nutrition status OR enteral nutrition) AND (point-of-care ultrasound). Relevant articles were reviewed, and keywords and index terms extracted from their titles and abstracts (e.g., preschool child, teenager, adolescent, critical illness, muscle, ultrasonography, etc.) were used to formulate a comprehensive search strategy for both PubMed and CNKI. This strategy can be adjusted and applied to various English and Chinese databases. Following this, a subsequent search was executed across all included databases using the complete set of identified MeSH terms, keywords, and index terms. The search was limited to papers published since establishment to 30 November 2025. Appendix A describes the full search strategies in all databases.

### 2.4 Study selection

All identified references were imported into EndNote X9 reference manager software. Records from different databases were merged, and duplicate entries were removed. A two-step screening procedure was implemented to select studies. The titles and abstracts of the studies were screened, followed by a full-text review. Two reviewers independently assessed the articles according to the eligibility criteria. Any disagreements were discussed, and a third reviewer was consulted if consensus could not be reached.

### 2.5 Data extraction

Using a structured data extraction form developed by the research group, two reviewers independently extracted relevant data from all included studies and recorded them in Microsoft Excel. Extracted data included: study design, study population, application of POCUS, and main results of the study. In addition, detailed implementation variables were extracted, including ultrasound model, transducer type, measurement site, body position, parameters, timing of assessment, operator, and methodological details. Any reviewer disagreements were addressed with an initial discussion phase, followed by final adjudication from a third reviewer if needed.

### 2.6 Quality appraisal

A basic appraisal of study quality was conducted to contextualize the strength of the evidence. Two reviewers independently assessed the methodological quality of the included studies using the appropriate JBI critical appraisal checklists based on study designs (34). Any discrepancies were resolved through discussion with a third reviewer. Given the scoping review methodology, the quality appraisal was intended solely to provide a background context for evidence strength rather than to serve as a criterion for study exclusion.

### 2.7 Synthesis of results

We performed a descriptive analysis of the data, consistent with the scoping review’s objective to investigate and describe the literature. The included studies were grouped and summarized based on the demographic characteristics of the pediatric patients, the purpose of POCUS application, key implementation steps, and outcomes. A comprehensive narrative summary was conducted to describe and categorize the current application status of POCUS in the nutrition assessment and enteral nutrition management of critically ill children across the included studies, highlighting trends, research gaps, and areas warranting further investigation. The grouping into three major domains (muscle mass assessment, feeding tube placement/positioning, and measurement of GRV and motility) was developed during data synthesis by grouping similar POCUS applications found across the included studies.

## 3 Results

### 3.1 Search results

**Figure 1:**
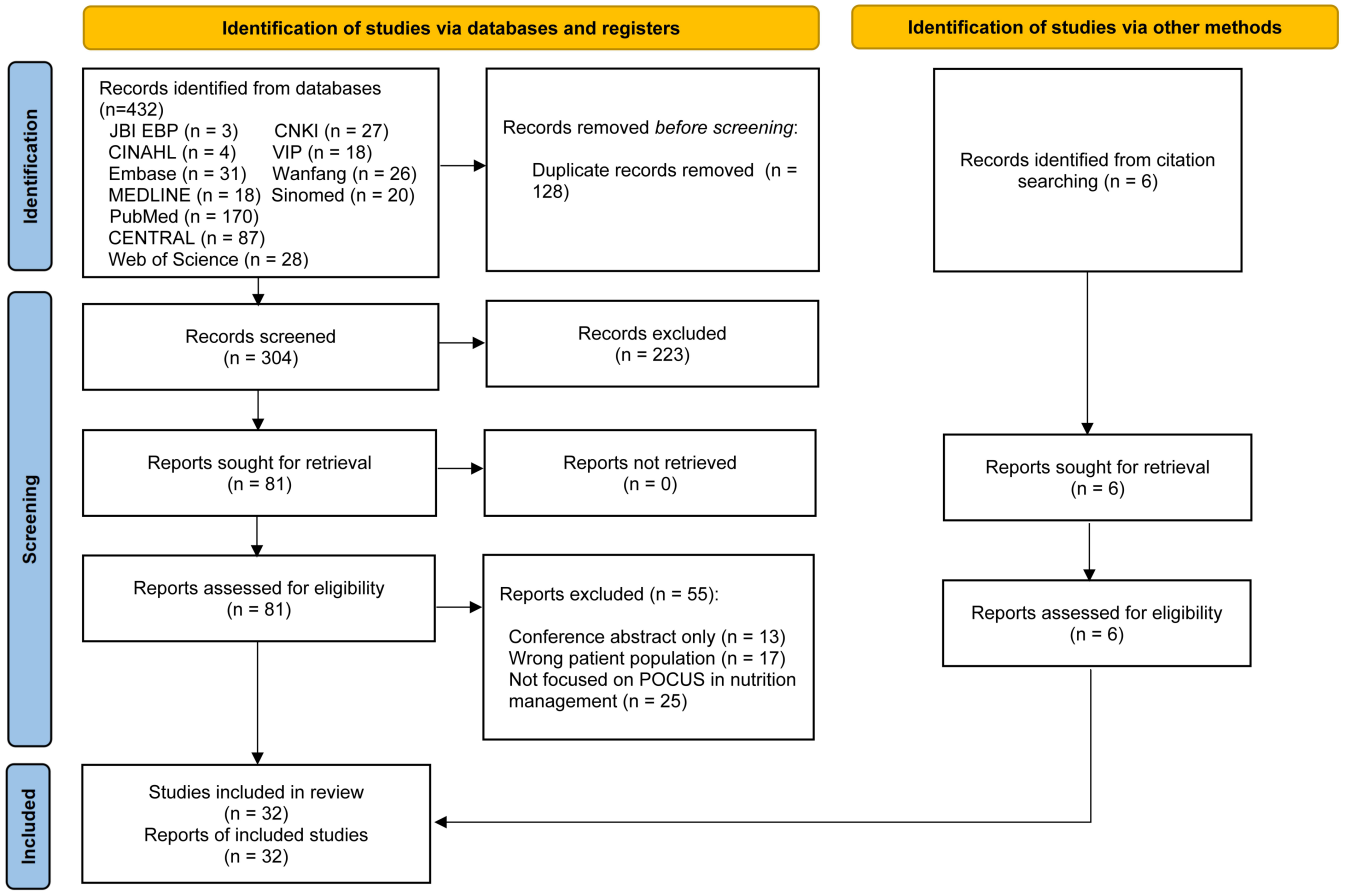
PRISMA flow diagram of study results. * Consider, if feasible to do so, reporting the number of records identified from each database or register searched (rather than the total number across all databases/registers). ** If automation tools were used, indicate how many records were excluded by a human and how many were excluded by automation tools. Adapted from Page et al. (73), licensed under CC BY 4.0.

The initial literature search identified 432 studies, 128 duplicates were excluded, and 304 for primary screening. After title and abstract review, 223 studies were excluded, resulting in 81 for further consideration. After reviewing full texts, 26 studies met the inclusion criteria. An additional 6 studies were identified from reference lists of included studies, resulting in a total of 32 studies were included in this scoping review. Reasons for full-text exclusion are presented in the PRISMA flow diagram (Figure 1).

### 3.2 Overview of studies

Characteristics of included studies are presented in Table 1. The United States conducted the most relevant research (10, 19, 35–39) (7/32, 21.9%), followed by Brazil (20, 40–43) (5/32, 15.6%), China (25, 44, 45) (3/32, 9.4%) and France (46–48) (3/32, 9.4%). The vast majority of the studies were published within the last 5 years, accounting for 65.6% (21/32) publications (19, 20, 23, 25, 36–43, 45, 47, 49–55), followed by 28.1% (9/32) studies (10, 26, 35, 46, 48, 56–59) published between 2015 and 2020. Most of the research consisted of primary studies, with prospective cohort studies being the most numerous, totaling 50% (16/32) studies (10, 20, 25, 37, 38, 43, 45–54). Among the primary studies, 25% (7/28) studies (36, 39, 40, 45, 55, 56, 60) had a sample size of less than 30, while 21.4% (6/28) studies (20, 23, 25, 38, 43, 58) had a sample size greater than or equal to 100. Of the 50% (16/32) included studies (10, 35, 37, 38, 40, 43, 44, 46–53, 60), the focus was on pediatric patients receiving mechanical ventilation, encompassing a total of 845 patients. The ages of children across the studies varied widely, spanning from 0.2 to 17 years, indicating significant heterogeneity. The quality appraisal results for each included study are summarized in Appendix B. Overall, the methodological quality of the included studies ranged from moderate to high.

**Table 1:** Overview and characteristics of included studies (*n* = 32).

Characteristic	Number of studies (*n*)	Percentage (%)
Country		
United States	7	21.9
Brazil	5	15.6
China	3	9.3
France	3	9.3
Japan	2	6.3
Spain	2	6.3
Canada	2	6.3
Singapore	2	6.3
United Kingdom	2	6.3
Egypt	1	3.1
India	1	3.1
Turkey	1	3.1
Belgium	1	3.1
Publication year		
Before 2010	1	3.1
2010–2014	1	3.1
2015–2020	9	28.1
2020–present	21	65.6
Study design		
Prospective cohort study	16	50
Diagnostic test accuracy study	6	18.8
Review	4	12.5
Cross-sectional study	3	9.4
Quasi-experimental research	1	3.1
Case report	1	3.1
Retrospective cohort study	1	3.1
Sample size (*n* = 28)		
X < 30	7	25.0
30 ≤ X < 50	8	28.6
50 ≤ X < 100	7	25.0
X ≥ 100	6	21.4

### 3.3 Application of POCUS in nutrition assessment and enteral nutrition management

**Figure 2:**
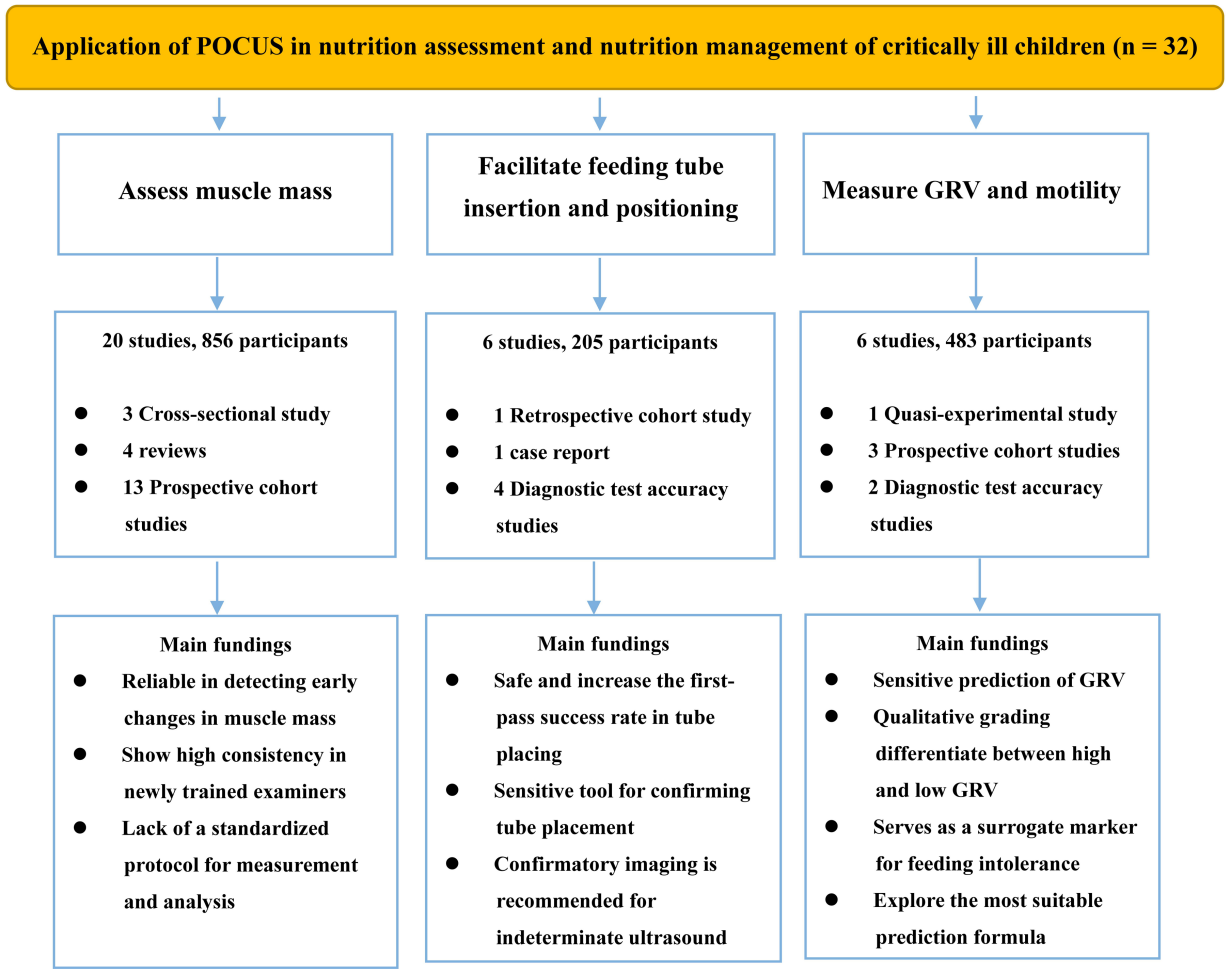
Application of POCUS in included studies.

Among all the included studies, POCUS was applied in the nutrition assessment and enteral nutrition management of critically ill children for the assessment of muscle mass, facilitation of feeding tube insertion and positioning, and measurement of GRV and motility (Figure 2).

#### 3.3.1 Assessment of muscle mass

**Figure 3:**
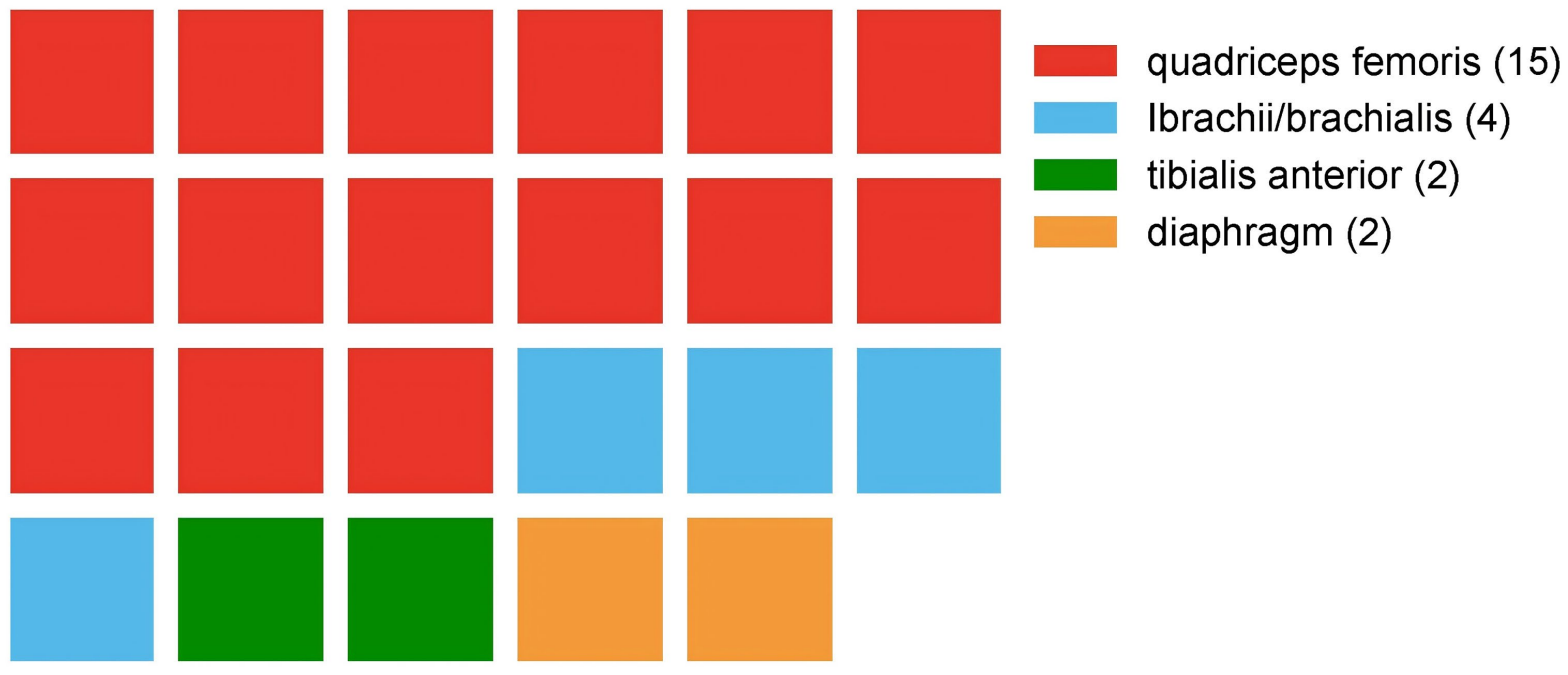
Muscles assessed in included studies.

Twenty studies (10, 19, 20, 26, 35, 37, 38, 40–43, 45, 46, 49–54, 57) involving 856 participants aimed to detect the early changes in muscle mass to monitor responses to nutritional interventions and dynamically evaluate nutritional risk. Detailed implementation characteristics of POCUS-based muscle assessment across the included studies are summarized in Table 2. Across the included studies, the quadriceps femoris was the most frequently assessed muscle, while the biceps brachii/brachialis and tibialis anterior were evaluated less commonly (Figure 3). Most studies measured muscle thickness, whereas cross-sectional area and echogenicity were reported in a smaller number of studies (37, 45, 52–54). Considerable heterogeneity was identified in muscle selection, anatomical landmarks, patient positioning, and scanning protocols. For quadriceps femoris assessment, measurement sites varied substantially across studies, with measurements obtained at different proportions of the distance between the anterior superior iliac spine (ASIS) and the superior border of the patella, including 2/5, 1/2, 3/5, and 2/3 of the thigh length (10, 20, 35, 37, 38, 40, 43, 49–54, 57), while some studies additionally selected the point of maximal muscle diameter (46, 53). Variations were also observed for tibialis anterior assessment, with measurements performed at either 1/4 or 1/3 of the distance between the inferior border of the patella and the lateral malleolus (10, 35). In contrast, the biceps brachii/brachialis was more consistently assessed at approximately 2/3 of the distance from the acromion to the antecubital crease (10, 35, 40, 43). For quadriceps femoris and tibialis anterior measurements, patients were commonly positioned supine with the lower limbs extended and relaxed (20, 35, 37, 38, 40, 43, 46, 49, 50, 52–54, 57), whereas upper limb assessments were generally performed with the arm extended and the forearm supinated (10, 35, 40, 43).

**Table 2:** Characteristics of POCUS applications for muscle mass assessment.

Study	USG model	Transducer	Timing	Operator	Patient position	Measured point	Methods	Main results
Robert (49)	LOGIQ-Q8/LOGIQe	Linear, 3.1-10MHz/4.2-13MHz	Repeated every 3 days until discharge from the ICU or until study day 15	Radiologists and physicians	Supine position Hip in extension, neutral adduction/abduction Patella rotated anteriorly	2/5 ASIS to superior patellar border	Target muscle: quadriceps femoris Measured parameter: thickness Minimize muscle compression Capture alternating short-axis and long-axis images Obtain two measurements in both axes	Quadriceps thickness measurement shows high reliability across different evaluators Fluid balance is not linked to changes in muscle thickness Achieving target calorie and protein intake is positively associated with muscle thickness change
Delia (50)	SonoSite	Linear, 12MHz	Within 24 h of initiating invasive MV and again at 72 h, 1 week, and weekly thereafter until extubation	PICU staff	Supine position Right leg extended and in neutral rotation	1/2 between the ASIS and the superior border of the patella	Target muscle: quadriceps femoris Measured parameter: thickness Minimize muscle compression Capture short-axis images Obtain three measurements	Over half of the children experienced muscle atrophy during their PICU stay
Frederic (46)	Vivid S6/SonoSite EDGE	Linear, 9-13Hz	Within the first 24 hours after PICU admission, between day 4 and day 5	PICU staff	Supine position Leg fully extended and in a neutral rotation	Widest portion of the thigh	Target muscle: quadriceps femoris Measured parameter: thickness Minimize muscle compression Capture short-axis and long-axis images Obtain two measurements in both axes	Intra-operator and Inter-operator repeatability for quadriceps thickness measurement was high Quadriceps muscle thickness decreased significantly during the PICU stay
Ruane (20)	Vicid Q	Linear, 3-13MHz	Within 72 h after PICU admission and then weekly until the 14th day of PICU stay	Sonographers	Supine position Knee in passive extension and neutral rotation	2/3 ASIS to the superior patella border	Target muscle: quadriceps femoris Measured parameter: thickness Maximize muscle compression Capture short-axis images Obtain three measurements	Quadriceps muscle thickness significantly decreased during the initial PICU stay The decrease in muscle thickness was associated with cumulative protein deficit, not with energy deficit
Jessica (40)	InnoSight	Linear, 4-12MHz	Within 24 h of intubation	Radiologists and physiotherapists	Supine position Arms and legs extended and muscles relaxed The upper extremity was supinated The lower extremity positioned with the knee extended and the ankle in a neutral position	Quadriceps femoris: 1/2 ASIS to the upper border of the patella Biceps brachii/brachialis: 2/3 the acromion to the antecubital fold	Target muscle: quadriceps femoris and biceps brachii/brachialis Measured parameter: thickness Minimize muscle compression Capture short-axis images Obtain three measurements	Intrarater and inter-rater reliability for the ultrasound measurements were high Measurement differences were small, with good agreement between assessors
Kay (35)	SonoSite Edge II	Linear, 13-6MHz	NA	Sonographers	Supine position Arms and legs extended with muscles relaxed Arms were supinated Knees extended and ankles placed in neutral position	Quadriceps femoris:1/2 ASIS to the superior aspect of the patella Biceps brachii/brachialis:2/3 acromion to the antecubital crease Tibialis anterior:1/4 inferior aspect of the patella to the lateral malleolus	Target muscle: quadriceps femoris, biceps brachii/brachialis, and tibialis anterior Measured parameter: thickness Minimize muscle compression Capture short-axis images Obtain three measurements	Expert and novice sonographers demonstrated similar levels of reliability in their measurements Ultrasound measurements for limb muscles showed better repeatability and consistency than those for the diaphragm
Jessica (43)	InnoSight	Linear, 4-12MHz	Within 24 h and 72 h after intubation and weekly until PICU discharge, limited to a maximum of 1 month	Radiologists and physiotherapists	Supine position Arms and legs extended with muscles relaxed Arms were supinated Knees extended and ankles placed in neutral position	Quadriceps femoral: 1/2 ASIS to the upper border of the patella biceps Brachii/brachialis: 2/3 the acromion to the antecubital fold	Target muscle: quadriceps femoris and biceps brachii/brachialis Measured parameter: thickness Minimize muscle compression Capture short-axis images Obtain three measurements	Significant muscle thickness loss occurred in both the biceps and quadriceps during the PICU stay POCUS is a valuable tool for monitoring muscle atrophy
Shereen (51)	General Electric	Linear, 11L Hz	On day 1 of admission, day 3, and day 7	Sonographers	N/A	1/2 ASIS to the upper border of the patella biceps	Target muscle: quadriceps femoris Measured parameter: thickness Maximize muscle compression Capture short-axis images Obtain three measurements	Quadriceps muscle thickness decreased significantly during the first week of PICU admission This muscle loss was correlated with clinical severity scores, nutritional intake, and mortality POCUS is a practical tool for assessing muscle wasting
Ryan (10)	SonoSite Edge II	Linear, 13-6MHz	At enrollment and at 5–8 days intervals until PICU discharge	PICU staff	N/A	Quadriceps femoris:1/2 ASIS to the superior aspect of the patella Biceps brachii/ brachialis:2/3 acromion to the antecubital crease Tibialis anterior:1/3 inferior aspect of the patella to the lateral malleolus	Target muscle: quadriceps femoris and biceps brachii/ brachialis Measured parameter: thickness Minimize muscle compression Capture short-axis images Obtain three measurements	Significant thickness loss occurred in the diaphragm and quadriceps, but not in the biceps or tibialis
Agam (52)	CX-50	Linear, 3-12MHz	On day 1 of admission, day 3, and day 7	Other investigators	Supine position leg extended and in neutral rotation	1/2 ASIS to the superior aspect of the patella	Target muscle: quadriceps femoris Measured parameter: thickness and echogenicity Minimize muscle compression Capture short-axis and long-axis images Obtain two measurements in both axes Echogenicity was measured using ImageJ software histogram function	Muscle thickness remained stable over 7 days, but rectus femoris echogenicity increased
Mohammed (37)	SonoSite X-Porte	Curvilinear, 2-5MHz	Immediately after they enrolled in the study, every 72-h intervals until extubation	Physiotherapists and clinical researchers	Supine position Limbs maintained in neutral alignment	2/5 of the femoral length, measured from the anterior superior iliac spine	Target muscle: quadriceps femoris Measured parameter: thickness and CSAMaintain consistent probe pressure Capture short-axis and long-axis images Obtain two measurements in both axes CSA was calculated using Sonosite’s area tool	A significant proportion of critically ill patients experienced muscle loss within 3-10 days of ICU admission and mechanical ventilation Lower protein intake relative to goals was associated with a higher likelihood of muscle loss
Lyvonne (53)	NA	NA	Day 1, 3, 5, 7 and 10 of PICU stay when possible	PICU staff	Supine position Leg fully extended and positioned in a neutral rotation	Widest portion of the thigh	Target muscle: quadriceps femoris Measured parameter: thickness and CSA Minimize muscle compression Capture short-axis and long-axis images Obtain two measurements in both axes	Universal muscle thinning occurred in critically ill children during PICU admission No clear link was found between this muscle thinning and early nutrition, illness severity, inflammation, or steroid use
Esther (38)	Venue Go	Linear, 4.2-13MHz	Repeated every 2 to 3 days until extubation	PICU staff	Supine position Arms and legs extended Feet in neutral position with regard to inversion and eversion	1/2 ASIS to the superior aspect of the patella	Target muscle: quadriceps femoris Measured parameter: thickness Minimize muscle compression Capture short-axis images Obtain two measurements	Muscle thickness showed a measurable daily change Achieving adequate caloric intake was associated with less muscle loss No significant correlation was found with mortality, illness severity, or several other clinical factors
Tom (57)	Vivid S6	Linear, 12MHz	NA	PICU staff	Supine position knee extended and the muscle relaxed	3/5 ASIS to the superior patellar border	Target muscle: quadriceps femoris Measured parameter: thickness Minimize muscle compression Capture short-axis images Obtain two measurements	Muscle thickness was lower and measurement variability was higher in children The accuracy of repeated measurements by the same observer was lower in children
Zhi (45)	LOGIQ-Q8	Linear	On the 1st, 4th and 7th day after admission to ICU	PICU staff	Supine position Head of the bed raised by 30° Lower limbs naturally straightened	1/2 ASIS to the superior aspect of the patella	Target muscle: rectus femoris, vastus intermedius Measured parameter: thickness and CSA Minimize muscle compression Capture short-axis images Obtain three measurements	Muscle ultrasound is a reliable monitoring tool in critically ill children Muscle atrophy is common and linked to inadequate nutrition and clinical severity
Chengsi (54)	LOGIQe	Linear, 5-13MHz	Within 48 hours of PICU admission, day 3, 7, and 10, at PICU and hospital discharge, and post-discharge	Other investigators	Supine position legs extended and neutral	1/2 ASIS to the superior aspect of the patella In children ≥6 years, 2/3 ASIS to the superior aspect of the patella	Target muscle: quadriceps femoris Measured parameter: thickness and echogenicity Minimize muscle compression Capture short-axis images Obtain three measurements Echogenicity was measured using ImageJ software	Muscle loss during the PICU stay was linked to inadequate energy intake

ASIS, anterior superior iliac spine; CSA, cross-sectional area.

For muscle mass assessment, linear probes (3–13 MHz) were used in most studies, while one study employed a curvilinear probe (2–5 MHz) (37). To minimize tissue deformation, studies commonly described the use of copious gel and minimal probe pressure during scanning, although two studies adopted a maximum compression protocol to reduce the influence of soft tissue compression or lower limb edema on measurement accuracy (20, 51). Measurements of quadriceps femoris muscle thickness were primarily obtained in the short-axis view, perpendicular to the femoral long axis, from the outer femoral cortex to the internal border of the muscle fascia, whereas studies assessing cross-sectional area commonly incorporated long-axis imaging by rotating the probe 90° from the short-axis view and positioning it over the thickest portion of the rectus femoris (46, 49, 52, 53). Most studies repeated measurements two to three times and used averaged values for analysis (10, 35, 38, 40, 43, 45, 50, 54, 57). Muscle echogenicity was assessed in two studies using the histogram function of ImageJ software (52, 54), and repeated POCUS assessments for longitudinal monitoring were typically performed every 3–7 days. Across studies, POCUS demonstrated generally high intra- and inter-operator reliability, with good agreement between expert and novice sonographers. Most studies reported significant muscle loss during PICU admission, particularly in the quadriceps femoris (46, 49), which was associated with inadequate nutritional intake and higher clinical severity.

#### 3.3.2 Facilitation of feeding tube insertion and positioning

Six studies (23, 36, 39, 55, 56, 60) involving 205 participants evaluated the application of POCUS in feeding tube insertion and positioning in critically ill children. Detailed implementation characteristics of POCUS in facilitating feeding tube insertion and positioning across the included studies are summarized in Table 3. Among these studies, POCUS was used to guide post-pyloric or nasogastric tube placement in two studies (23, 55), while the remaining studies applied POCUS to confirm the position of nasogastric, orogastric, or post-pyloric feeding tubes (36, 39, 56, 60). All studies used abdominal ultrasonography of the gastric antrum to assess tube location, and several additionally incorporated cervical ultrasonography to confirm esophageal tube passage (36, 55, 56). Patients were generally positioned supine during assessment, whereas right lateral decubitus positioning was used during post-pyloric tube placement to facilitate pyloric passage (23). Considerable variation was observed in ultrasound equipment and scanning protocols. Convex probes were most commonly used for gastric imaging, whereas linear probes were used for cervical assessment and in younger children or those with limited subcutaneous tissue (36, 48, 56, 58). Across studies, feeding tube localization was primarily based on the identification of two parallel hyperechoic lines. Different scanning strategies were reported, including continuous tracking along the tube pathway (36) and targeted assessment at key anatomical landmarks (39, 56). To improve visualization during tube confirmation, some studies additionally used air bolus or air-saline injection into the stomach to generate hyperechoic gastric fog when direct visualization was difficult (39).

**Table 3:** Characteristics of POCUS applications in facilitating feeding tube insertion and positioning.

Study	USG model	Transducer	Timing	Operator	Reference standard	Methods	Main results
Ichiro (23)	SonoSite Edge	Linear	NA	PICU staff	X-ray	POCUS guided placement of a post-pyloric feeding tube was divided into four-steps Confirm final tube tip position within the duodenum distal to the superior mesenteric artery	The first-pass success rate for postpyloric feeding tube placement was significantly higher with ultrasound guidance The procedure was safe, with no reported complications or adverse events
Alonso (36)	SonoSite PX	Curvilinear, 10-3MHz; Linear, 19-5MHz	After the bedside nurse placed the enteral feeding tube	PICU staff	X-ray Identifying gastric tubes: sensitivity 85.7%, specificity 57.1% Identifying postpyloric tubes: sensitivity 66.7% specificity 87.5%	An abdominal radiograph was obtained to confirm the tube placement limited to 5 min A subsequent 2-min cervical examination performed only if the feeding tube was not initially visualized	POCUS is a safe and rapid method for localizing feeding tubes in the PICU The technique demonstrated a high success rate and reasonable procedure time No complications were reported during the POCUS-guided placement procedures
Yunus (56)	Acuson X300	Linear, 8 MHz; curvilinear, 5MHz	After the insertion of NGT	PICU staff	X-ray Identifying nasogastric tubes: sensitivity 100%,	POCUS confirmed NGT placement via transverse and longitudinal neck scans and epigastric visualization at the esophagogastric junction Injection of an air-saline mixture to produce a hyperechoic fog in the stomach if direct tip visualization failed	POCUS is an effective and sensitive method for verifying correct nasogastric tube placement
Mary (39)	Philips Sqarq	Curvilinear, 5-1MHz/4-2MHz	After tube placement and when possible before X-ray confirmation	PICU staff	X-ray Identifying nasogastric and orogastric tubes: sensitivity 88%,	A standardized abdominal ultrasound scanning protocol was utilized to identify the tube The entire procedure limited to a maximum of 10 min	POCUS effectively verified feeding tube position in the PICU The technique demonstrated reliable sensitivity
Edward (55)	CX50	Linear, 7-12MHz; curvilinear, 2-5MHz	A nurse inserted the NGT	Anesthesiologists	N/A	Standardized scanning protocols were performed to visualize the gastric antrum and body, the thyroid, carotid artery, trachea, and esophagus	POCUS can assist in pediatric NGT placement and help reduce suboptimal positioning Injecting an air bolus may improve visualization in the stomach, but further studies are needed to enhance success rates
Flores (60)	N/A	N/A	Before initiating enteral nutrition	PICU staff	N/A	N/A	Abdominal ultrasound indicated that the feeding tube was within the intestinal lumen; however, this misinterpretation led to feeding administration and subsequent development of severe peritonitis.

Overall, POCUS demonstrated promising utility in facilitating feeding tube insertion and positioning. Ultrasound-guided techniques were associated with high first-pass success rates, reliable sensitivity for tube localization and position verification, and reasonable procedure times. Compared with X-ray confirmation, POCUS demonstrated sensitivities ranging from 85.7 to 100% for gastric tube placement and 66.7% for post-pyloric tube placement (36, 39, 56). Several studies also reported that POCUS-assisted techniques could reduce suboptimal tube positioning. Most studies reported no procedure-related complications or adverse events, supporting the feasibility and safety of POCUS-guided feeding tube management in the PICU setting (23, 36, 55). However, one case report described an intestinal perforation that was not promptly recognized because of misinterpretation of POCUS findings during post-pyloric tube localization, leading to severe intra-abdominal infection after enteral feeding (60). This finding highlights the importance of operator experience and standardized interpretation protocols when applying POCUS for feeding tube confirmation.

#### 3.3.3 Measurement of GRV and motility

Across 6 studies (25, 44, 47, 48, 58, 59) involving 483 patients, POCUS was used to assess GRV and gastric motility, primarily through gastric antral CSA measurements. Detailed implementation characteristics of POCUS in measurement of GRV and motility are summarized in Table 4. Most studies performed CSA assessment in the right lateral decubitus (RLD) position and applied regression-based formulas to estimate GRV, with the most commonly used model incorporating RLD CSA and age (47, 48, 58). One study used a modified protocol combining RLD positioning with head-of-bed elevation and an alternative formula integrating CSA with qualitative grading (25), whereas another performed CSA measurements in the supine position and demonstrated a positive linear correlation between CSA and GRV (59). Serial ultrasound assessment of gastric antral changes was also used to dynamically evaluate gastric emptying and gastric motility during enteral feeding.

**Table 4:** Characteristics of POCUS applications in measurement of GRV and motility.

Study	USG model	Transducer	Timing	Operator	GRV calculation method	Patient position	Methods	Main results
Jinjiu (25)	NA	Curvilinear, 2–5 Hz; linear, 5–12 Hz	Within 10 min prior to each feeding	PICU staff	GRV (mL) = −12.9 + 10.3 (RLD CSA [cm^2^]) + 3.3 × Grade 1 + 10.1 × Grade 2	Supine with the bed elevated at 45° Right lateral recumbent position	A CSA was obtained in the supine position and right lateral recumbent position Measuring the gastric antrum CSA using the tracing method to delineate the outer gastric wall Obtain three measurements	Ultrasound measurements strongly correlated with GRV in children The model showed high agreement between predicted and actual GRV values
Frederic (47)	Vivid S6 Vivid S70N	Curvilinear, 2–9/4 Hz; linear, 9 Hz	Prior to sedation	PICU staff	GRV (mL) = −7.8 + (3.5 × RLD CSA [cm^2^]) + (0.127 × age [months])	First in a supine position Then in an RLD position	Measurements of gastric antrum larger and shorter diameters were performed first in supine position and then in an RLD position Three measurements were performed in both positions	No association was found between GRV or sonographic gastric volume and clinical signs of feeding intolerance
Yuan (44)	CX50	Curvilinear, 3.5MHz	Every 4 h	PICU staff	N/A	RLD position	Measure the amplitude of the gastric antrum contraction and the frequency of the gastric antrum contraction within 3 minutes	Ultrasound-guided feeding management was associated with faster achievement of nutritional targets, higher caloric intake, and improved nutritional biomarkers
Desgranges (48)	S-Nerve	Linear, 10-13MHz; curvilinear, 2-5MHz	On arrival in the operating theatre, before induction of anaesthesia; and after the end of surgery, before awakening from anaesthesia and tracheal extubation	PICU staff	GRV (ml/kg) = [7.8 + 0.035 * CSA (mm^2^) + 0.127 * age (months)]/body weight (kg)	First in a supine position Then in an RLD position	Measurements of gastric antrum larger and shorter diameters were performed in an RLD position Qualitative assessment of the stomach antrum contents was also performed using a 3-point qualitative grading scale	Gastric volume remained low and consistent before and after elective ENT surgery in children
Adam (58)	CX50	Curvilinear, 2-5MHz; linear, 7-12MHz	After induction of anesthesia	Anesthesiologists	GRV (mL) = −7.8 + (3.5 × RLD CSA [cm^2^]) + (0.127 × age [months])	First in a supine position Then in an RLD position	CSA was measured with a free-tracing method using the internal caliper of the ultrasound unit Obtain three measurements	POCUS assessment of the gastric antrum provides useful information regarding gastric content and volume in pediatric patients
Chikako (59)	Venue 40	Curvilinear, 3.32MHz;	Before general anesthesia induction	Anesthesiologists	N/A	Supine position	Supine CSA was measured without waiting for the gastric antrum to be at rest between peristaltic contractions Obtain only one measurement	Gastric antrum cross-sectional area measured by ultrasound correlated with aspirated gastric fluid volume in fasted pediatric patients The ultrasound method is applicable for assessing minimal gastric contents in children

GRV, gastric residual volume; RLD, right lateral decubitus; CSA, cross-sectional area.

Considerable methodological heterogeneity was identified across studies. CSA was measured either by manual tracing of the gastric antrum outer wall or by geometric estimation based on anteroposterior and craniocaudal diameters (25, 48, 58). Several studies performed repeated measurements and used averaged values to improve reliability. In addition, some studies incorporated a semi-quantitative grading system (Grades 0–2) based on gastric fluid visibility in supine and RLD positions to complement quantitative CSA assessment (25, 48, 58). Ultrasound-derived gastric measurements demonstrated good correlation and agreement with aspirated gastric volume, supporting the feasibility of POCUS as a non-invasive bedside method for estimating gastric contents, including minimal residual volumes (25, 39, 58). Clinically, ultrasound-guided feeding management was associated with faster achievement of nutritional targets, higher caloric intake, and improved nutritional biomarkers (44). However, no consistent association was identified between sonographic or measured GRV and clinical signs of feeding intolerance (47), and the optimal prediction model for GRV estimation remains to be established.

## 4 Discussion

POCUS plays a significant role in the nutrition management of critically ill children. Findings from this scoping review indicate that most of the studies (21/32) were published within the last 5 years, suggesting this field of research is still in its initial stage. The application of POCUS in this field primarily focuses on three areas: muscle mass assessment, facilitating feeding tube placement and confirming tip position, and evaluating gastric motility along with measuring GRV. Although POCUS demonstrated promising utility across muscle assessment, feeding tube management, and gastric monitoring, substantial variability in study protocols and outcome measures remains a common challenge across the current evidence base.

### 4.1 Principal findings

#### 4.1.1 Muscle mass assessment

This review underscores the emerging role of POCUS as a tool for objective muscle assessment in critically ill children while highlighting important methodological limitations related to anatomical variability and measurement techniques. Compared with traditional indicators such as body weight and serum albumin, quadriceps muscle ultrasound appears less influenced by fluid shifts or medication use and may provide a more sensitive reflection of short-term changes in nutritional adequacy (64–66). These findings support the potential value of POCUS for real-time monitoring of nutrition-related muscle changes in critically ill children. However, considerable methodological heterogeneity remains across studies. As the quadriceps is a spindle-shaped muscle with variable thickness along its length, there is no consensus on optimal measurement sites (32). Variability also exists in measured parameter, number of repeated acquisitions and probe pressure techniques (46, 50, 61). These methodological inconsistencies limit comparability across studies and hinder the establishment of standardized, reproducible protocols for clinical use. Furthermore, pediatric-specific factors add additional complexity to the feasibility of quadriceps ultrasound. Young age, limited cooperation, and sedation status may affect measurement consistency and practical implementation in clinical settings (43, 62–64). These factors should be considered when interpreting ultrasound-based muscle assessments in critically ill children.

#### 4.1.2 Facilitating feeding tube placement and confirming tip position

Current evidence supports the feasibility of POCUS for guiding feeding tube placement in pediatric patients, highlighting its distinct advantage in reducing radiation exposure. Compared with adult protocols, pediatric studies more commonly rely on patient positioning and saline enhancement to facilitate tube visualization without pharmacologic agents (23, 65). High first-attempt success rates reported across studies further suggest that POCUS may serve as a useful bedside adjunct for enteral access (23). Beyond procedural feasibility, an important potential advantage of POCUS is the reduction in radiation exposure associated with repeated radiographic confirmation, which may be particularly beneficial for critically ill children requiring prolonged PICU care (36). In addition, bedside ultrasound may improve the timeliness of feeding management by avoiding delays related to radiographic examination and patient transport (39). Although scanning protocols varied among studies, identification of two parallel hyperechoic lines was consistently used for tube localization and demonstrated good sensitivity compared with radiography (36, 39, 56). Nevertheless, several pediatric-specific factors, including recent feeding, crying, and excessive air swallowing, may reduce image quality and complicate interpretation (39). Moreover, a reported case of severe intra-abdominal infection caused by misinterpretation of tube position highlights that POCUS should be applied cautiously and, when necessary, combined with complementary confirmation methods to ensure patient safety (60, 66).

#### 4.1.3 Measuring GRV and evaluating gastric motility

POCUS shows potential for gastric monitoring in critically ill children, but current evidence is constrained by the absence of validated GRV prediction models specific to this population and limited data on gastric motility assessment. Existing GRV estimation formulas were largely derived from fasting pediatric populations before endoscopy (58), raising concerns regarding their applicability in critically ill children. One study developed a GRV estimation model specifically for critically ill pediatric patients using right lateral decubitus (RLD) gastric antral cross-sectional area (CSA) and qualitative grading scores (25), although further validation is still needed. Variability in patient positioning, CSA measurement methods, and repeated scanning protocols across studies may further affect measurement consistency and limit comparability (25, 47, 48, 58, 59). While the RLD position is generally considered optimal for gastric volume estimation (67), maintaining this position and assessing gastric motility may be difficult in awake or uncooperative children. A simplified supine scanning protocol has therefore been proposed and demonstrated a positive correlation between gastric antral CSA and aspirated gastric volume (59), suggesting potential feasibility in selected pediatric settings. Compared with adults, evidence regarding ultrasound assessment of gastric motility in children remains limited (44, 68), highlighting the need for further pediatric-specific validation and protocol development.

### 4.2 Implications for future clinical practice and research

POCUS may serve as a practical bedside adjunct for nutritional management in critically ill children by enabling repeatable muscle assessment, feeding tube guidance, and gastric monitoring while potentially reducing radiation exposure and delays related to radiographic confirmation. In resource-limited settings, POCUS may be particularly valuable due to the portability of ultrasound devices and reduced dependence on radiology infrastructure, patient transport, and repeated imaging examinations, thereby improving accessibility of bedside diagnostic support (69). However, successful clinical implementation requires not only simplified pediatric scanning protocols but also structured, competency-based training and supervision to ensure reliable interpretation and patient safety, given the inherently operator-dependent nature of POCUS.

Future research should focus on establishing standardized pediatric-specific POCUS protocols, including consensus on muscle measurement techniques and gastric assessment methods. Multicenter validation studies are needed to determine the clinical reliability and prognostic value of ultrasound-based nutritional monitoring. Further work should also evaluate implementation feasibility, cost-effectiveness, and scalable training frameworks across different healthcare settings, particularly in low-resource PICU environments.

### 4.3 Strengths and limitations

Strengths of this study include its rigorous methodological approach, which adhered to the PRISMA-ScR reporting framework and featured prospective protocol registration on the Open Science Framework. This methodological rigor and transparency serve as a reference model for future exploratory research on similar clinical technologies. A comprehensive search strategy enabled a thorough and critical synthesis of the current evidence across various studies applying POCUS in the nutrition management of critically ill children. By systematically organizing and comparing the existing evidence, this review has preliminarily distilled core operational elements and a foundational training framework, thereby providing a conceptual basis for establishing standardized operational or training pathways in the future.

This scoping review has several limitations. Many included studies were small-sample, single-center exploratory investigations with substantial heterogeneity in patient populations, ultrasound protocols, and outcome definitions, introducing potential selection bias and limiting the generalizability and comparability of findings across PICU settings. In addition, the current evidence base is predominantly observational, with a lack of randomized controlled trials evaluating the effectiveness and clinical impact of POCUS-guided nutritional assessment and feeding management. Furthermore, most studies did not report long-term or patient-centered outcomes, limiting evaluation of the sustained clinical relevance of POCUS-based interventions. These limitations highlight the need for future large-scale, multicenter, and methodologically rigorous studies to support standardized clinical implementation.

## 5 Conclusion

The current evidence suggests that POCUS has potential applications in the nutrition management of critically ill children, particularly for muscle mass assessment, feeding tube placement assistance, and gastric monitoring. Quadriceps ultrasound was the most frequently studied approach for muscle assessment, while ultrasound-guided feeding tube localization demonstrated the potential to reduce radiation exposure and improve bedside feeding management. However, considerable heterogeneity exists across studies regarding scanning protocols, measurement techniques, and outcome definitions, limiting comparability and clinical standardization. In addition, the evidence base remains largely observational and exploratory. Future multicenter studies are needed to validate pediatric-specific protocols and clarify the clinical impact of POCUS-guided nutritional management in critically ill children.
