# Supplementary material for: Point-of-care ultrasound in nutrition assessment and enteral nutrition management of critically ill children: a scoping review
Source: Front Nutr. 2026 Jun 22;13:1849240. doi: 10.3389/fnut.2026.1849240 (PMC13333426; doi:10.3389/fnut.2026.1849240)
Supplement: Supplementary file 1 [file Table_1.DOC]

Appendix II: Search strategy

Pubmed

Data searched: December 19, 2025

Limites: English language, from database inception to November 30, 2025

| # | Search terms | Result retrieved |
| --- | --- | --- |
| 1 | (ultrasonography[MeSH Terms]) | 518047 |
| 2 | (Ultrasonic Tomography OR Ultrasound Imaging OR Point-of-Care Ultrasound OR bedside ultrasound[Title/Abstract]) | 657966 |
| 3 | 1OR 2 | 657966 |
| 4 | (child OR child,preschool OR adolescent OR infant OR pediatrics[MeSH Terms]) | 5276642 |
| 5 | (children OR preschool child* OR adolescence OR teen* OR youth* OR infants OR paediatric*[Title/Abstract]) | 5323315 |
| 6 | 4 OR 5 | 5335186 |
| 7 | (critical care nursing OR critical illness OR intensive care units, pediatric[MeSH Terms]) | 163180 |
| 8 | (intensive care nursing OR critically ill OR pediatric ICU OR pediatric intensive care unit*[Title/Abstract]) | 229237 |
| 9 | 7 OR 8 | 229237 |
| 10 | (nutritional status OR nutrition assessment OR enteral nutrition OR parenteral nutrition OR muscle OR Muscle, Skeletal[MeSH Terms]) | 1565007 |
| 11 | ((nutrition index* OR prognostic nutritional index OR mini nutrition assessment OR enteral feeding OR force feeding[Title/Abstract])) OR (tube feeding OR Gastric retention volume OR Gastric retention OR skeletal muscle* OR body composition[Title/Abstract]) | 1122497 |
| 12 | 10 OR 11 | 2169819 |
| 13 | 3 AND 6 AND 9 AND 12 | 170 |
| 14 | From database inception to November 30, 2025 | 170 |

Embase

Data searched: December 19, 2025

Limites: English language, from database inception to November 30, 2025

| # | Search terms | Result retrieved |
| --- | --- | --- |
| 1 | 'ultrasonic tomography':ab,ti OR 'ultrasound imaging':ab,ti OR 'point-of-care ultrasound':ab,ti OR 'bedside ultrasound':ab,ti OR ultrasonography:ab,ti | 191246 |
| 2 | child:ab,ti OR adolescent:ab,ti OR infant:ab,ti OR pediatrics:ab,ti OR children:ab,ti OR 'preschool child*':ab,ti OR adolescence:ab,ti OR teen*:ab,ti OR youth*:ab,ti OR infants:ab,ti OR paediatric*:ab,ti | 3037629 |
| 3 | 'critical care nursing':ab,ti OR 'critical illness':ab,ti OR 'intensive care nursing':ab,ti OR 'critically ill':ab,ti OR 'pediatric icu':ab,ti OR 'pediatric intensive care unit*':ab,ti | 138661 |
| 4 | 'nutritional status':ab,ti OR 'nutrition assessment':ab,ti OR 'enteral nutrition':ab,ti OR 'parenteral nutrition':ab,ti OR muscle:ab,ti OR 'nutrition index*':ab,ti OR 'prognostic nutritional index':ab,ti OR 'mini nutrition assessment':ab,ti OR 'enteral feeding':ab,ti OR 'force feeding':ab,ti OR 'tube feeding':ab,ti OR 'gastric retention volume':ab,ti OR 'gastric retention':ab,ti OR 'skeletal muscle*':ab,ti OR 'body composition':ab,ti | 1275169 |
| 5 | 1 AND 2 AND 3 AND 4 | 31 |
| 6 | From database inception to November 30, 2025 | 31 |

MEDLINE

Data searched: December 19, 2025

Limites: English language, from database inception to November 30, 2025

| # | Search terms | Result retrieved |
| --- | --- | --- |
| 1 | (Ultrasonic Tomography or Ultrasound Imaging or Point-of-Care Ultrasound or bedside ultrasound or ultrasonography).ab. | 119253 |
| 2 | (child or adolescent or infant or pediatrics or children or preschool child* or adolescence or teen* or youth* or infants or paediatric*).ab. | 1808312 |
| 3 | (critical care nursing or critical illness or intensive care nursing or critically ill or pediatric ICU or pediatric intensive care unit*).ab. | 76346 |
| 4 | (nutritional status or nutrition assessment or enteral nutrition or parenteral nutrition or muscle or nutrition index* or prognostic nutritional index or mini nutrition assessment or enteral feeding or force feeding or tube feeding or Gastric retention volume or Gastric retention or skeletal muscle* or body composition).ab. | 859350 |
| 5 | 1 AND 2 AND 3 AND 4 | 18 |
| 6 | From database inception to November 30, 2025 | 18 |

Web of Science

Data searched: December 19, 2025

Limites: English language, from database inception to November 30, 2025

| # | Search terms | Result retrieved |
| --- | --- | --- |
| 1 | Ultrasonic Tomography or Ultrasound Imaging or Point-of-Care Ultrasound or bedside ultrasound or ultrasonography (Abstract) | 196781 |
| 2 | child OR adolescent OR infant OR pediatrics OR children OR preschool child* OR adolescence OR teen* OR youth* OR infants OR paediatric*(Abstract) | 2001320 |
| 3 | critical care nursing OR critical illness OR intensive care nursing OR critically ill OR pediatric ICU OR pediatric intensive care unit* (Abstract) | 113580 |
| 4 | nutritional status OR nutrition assessment OR enteral nutrition OR parenteral nutrition OR muscle OR nutrition index* OR prognostic nutritional index OR mini nutrition assessment OR enteral feeding OR force feeding OR tube feeding OR Gastric retention volume OR Gastric retention OR skeletal muscle* OR body composition (Abstract) | 1091230 |
| 5 | 1 AND 2 AND 3 AND 4 | 28 |
| 6 | From database inception to November 30, 2025 | 28 |

Cochrane Central Register of Controlled Trials

Data searched: December 19, 2025

Limites: English language, from database inception to November 30, 2025

| # | Search terms | Result retrieved |
| --- | --- | --- |
| 1 | Ultrasonic Tomography OR Ultrasound Imaging OR Point-of-Care Ultrasound OR bedside ultrasound | 16671 |
| 2 | Mesh descriptor: [ultrasonography] exploded all trees | 19742 |
| 3 | #1 OR #2 | 29713 |
| 4 | Mesh descriptor: [child] exploded all trees | 84008 |
| 5 | Mesh descriptor: [pediatrics] exploded all trees | 1075 |
| 6 | Mesh descriptor: [adolescent] exploded all trees | 139384 |
| 7 | Mesh descriptor: [infant] exploded all trees | 46558 |
| 8 | children OR preschool child* OR adolescence OR teen* OR youth* OR infants OR paediatric* | 262335 |
| 9 | #4 OR #5 OR #6 OR #7 OR #8 | 358775 |
| 10 | Mesh descriptor: [Critical Care Nursing] exploded all trees | 93 |
| 11 | Mesh descriptor: [Critical Illness] exploded all trees | 3929 |
| 12 | Mesh descriptor: [Intensive Care Units, Pediatric] exploded all trees | 1859 |
| 13 | intensive care nursing OR critically ill OR pediatric ICU OR pediatric intensive care unit* | 18301 |
| 14 | #10 OR #11 OR #12 OR #13 | 20027 |
| 15 | Mesh descriptor: [Nutritional Status] exploded all trees | 3807 |
| 16 | Mesh descriptor: [Nutrition Assessment] exploded all trees | 986 |
| 17 | Mesh descriptor: [Enteral Nutrition] exploded all trees | 2540 |
| 18 | Mesh descriptor: [Muscles] exploded all trees | 22816 |
| 19 | nutrition index* OR prognostic nutritional index OR mini nutrition assessment OR enteral feeding OR force feeding OR tube feeding OR intravenous feeding* OR parenteral feeding* OR Gastric retention volume OR Gastric retention OR skeletal muscle* OR body composition | 61151 |
| 20 | #15 OR #16 OR #17 OR #18 OR #19 | 76554 |
| 21 | #3 AND #9 AND #14 AND #20 | 87 |
| 22 | From database inception to November 30, 2025 | 87 |

JBI Best Practice

Data searched: December 19, 2025

Limites: English language, from database inception to November 30, 2025

| # | Search terms | Result retrieved |
| --- | --- | --- |
| 1 | (Ultrasonic Tomography or Ultrasound Imaging or Point-of-Care Ultrasound or bedside ultrasound or ultrasonography).af. | 89 |
| 2 | (child or adolescent or infant or pediatrics or children or preschool child* or adolescence or teen* or youth* or infants or paediatric*).af. | 2715 |
| 3 | (critical care nursing or critical illness or intensive care nursing or critically ill or pediatric ICU or pediatric intensive care unit*).af. | 465 |
| 4 | (nutritional status or nutrition assessment or enteral nutrition or parenteral nutrition or muscle or nutrition index* or prognostic nutritional index or mini nutrition assessment or enteral feeding or force feeding or tube feeding or Gastric retention volume or Gastric retention or skeletal muscle* or body composition).af. | 1206 |
| 5 | 1 AND 2 AND 3 AND 4 | 3 |
| 6 | From database inception to November 30, 2025 | 3 |

CINAHL

Data searched: December 19, 2025

Limites: English language, from database inception to November 30, 2025

| # | Search terms | Result retrieved |
| --- | --- | --- |
| 1 | AB (Ultrasonic Tomography OR Ultrasound Imaging OR Point-of-Care Ultrasound OR bedside ultrasound OR ultrasonography) | 26010 |
| 2 | AB (child OR adolescent OR infant OR pediatrics OR children OR preschool child* OR adolescence OR teen* OR youth* OR infants OR paediatric*) | 634038 |
| 3 | AB (critical care nursing OR critical illness OR intensive care nursing OR critically ill OR pediatric ICU OR pediatric intensive care unit*) | 32290 |
| 4 | AB (nutritional status OR nutrition assessment OR enteral nutrition OR parenteral nutrition OR muscle OR nutrition index* OR prognostic nutritional index OR mini nutrition assessment OR enteral feeding OR force feeding OR tube feeding OR Gastric retention volume OR Gastric retention OR skeletal muscle* OR body composition） | 256461 |
| 5 | 1 AND 2 AND 3 AND 4 | 4 |
| 6 | From database inception to November 30, 2025 | 4 |

CNKI

Data searched: December 19, 2025

Limites: Chinese language，from database inception to November 30, 2025

| # | Search terms | Result retrieved |
| --- | --- | --- |
| 1 | （篇关摘：超声检查 + 超声诊断 + 超声成像 + 即时超声 + 床旁超声 + 超声(精确)） | 628016 |
| 2 | （篇关摘：儿童 + 青少年 + 婴儿 + 儿科学 + 小儿 + 学龄前儿童 + 少年 + 婴幼儿 + 患儿(精确)） | 1842869 |
| 3 | （篇关摘：危重症护理 + 危重病 + 重症监护病房 + ICU + PICU (精确)） | 129335 |
| 4 | （篇关摘：营养状况 + 营养评价 + 肠道营养 + 胃肠外营养 + 肌肉 + 骨骼肌 + 营养指标 + 营养评估 + 微型营养评价 + 预后营养指数 + 肠内营养 + 鼻饲 + 管饲 + 十二指肠管 + 胃残余量(精确)）OR（篇关摘：胃潴留 + 喂养不耐受 + 身体成分(精确)） | 394412 |
| 5 | 1 AND 2 AND 3 AND 4 | 27 |

VIP

Data searched: December 19, 2025

Limites: Chinese language，from database inception to November 30, 2025

| # | Search terms | Result retrieved |
| --- | --- | --- |
| 1 | 摘要=超声检查 OR 超声诊断 OR 超声成像 OR 即时超声 OR 床旁超声 OR 超声 | 480991 |
| 2 | 摘要=儿童 OR 青少年 OR 婴儿 OR 儿科学 OR 小儿 OR 学龄前儿童 OR 少年 OR 婴幼儿 OR 患儿 | 1375132 |
| 3 | 摘要=危重症护理 OR 危重病 OR 重症监护病房 OR ICU OR PICU | 106530 |
| 4 | 摘要=营养状况 OR 营养评价 OR 肠道营养 OR 胃肠外营养 OR 肌肉 OR 骨骼肌 OR 营养指标 OR 营养评估 OR 微型营养评价 OR 预后营养指数 OR 肠内营养 OR 鼻饲 OR 管饲 OR 十二指肠管OR 胃残余量 OR 胃潴留 OR 喂养不耐受 OR 身体成分 | 356120 |
| 5 | 1 AND 2 AND 3 AND 4 | 18 |

WanFang

Data searched: December 19, 2025

Limites: Chinese language，from database inception to November 30, 2025

| # | Search terms | Result retrieved |
| --- | --- | --- |
| 1 | 摘要:(超声检查 OR 超声诊断 OR 超声成像 OR 即时超声 OR 床旁超声 OR 超声) | 686070 |
| 2 | 摘要:(儿童 OR 青少年 OR 婴儿 OR 儿科学 OR 小儿 OR 学龄前儿童 OR 少年 OR 婴幼儿 OR 患儿) | 2046789 |
| 3 | 摘要:(危重症护理 OR 危重病 OR 重症监护病房 OR ICU OR PICU) | 215567 |
| 4 | 摘要:(营养状况 OR 营养评价 OR 肠道营养 OR 胃肠外营养 OR 肌肉 OR 骨骼肌 OR 营养指标 OR 营养评估 OR 微型营养评价 OR 预后营养指数 OR 肠内营养 OR 鼻饲 OR 管饲 OR 十二指肠管OR 胃残余量 OR 胃潴留 OR 喂养不耐受 OR 身体成分) | 611143 |
| 5 | 1 AND 2 AND 3 AND 4 | 20 |

SinoMed

Data searched: December 19, 2025

Limites: Chinese language，from database inception to November 30, 2025

| # | Search terms | Result retrieved |
| --- | --- | --- |
| 1 | ( "超声检查"[摘要:智能] OR "超声诊断"[摘要:智能] OR "超声成像"[摘要:智能] OR "即时超声"[摘要:智能] OR "床旁超声"[摘要:智能] OR "超声"[摘要:智能]) | 472946 |
| 2 | ( "儿童"[摘要:智能] OR "青少年"[摘要:智能] OR "婴儿"[摘要:智能] OR "儿科学"[摘要:智能] OR "小儿"[摘要:智能] OR "学龄前儿童"[摘要:智能] OR "少年"[摘要:智能] OR "婴幼儿"[摘要:智能] OR "患儿"[摘要:智能]) | 2412368 |
| 3 | ( "危重症护理"[摘要:智能] OR "危重病"[摘要:智能] OR "重症监护病房"[摘要:智能] OR "ICU"[摘要:智能] OR "PICU"[摘要:智能]) | 296847 |
| 4 | ( "营养状况"[常用字段:智能] OR "营养评价"[常用字段:智能] OR "肠道营养"[常用字段:智能] OR "胃肠外营养"[常用字段:智能] OR "肌肉"[常用字段:智能] OR "骨骼肌"[常用字段:智能] OR "营养指标"[常用字段:智能] OR "营养评估"[常用字段:智能] OR "微型营养评价"[常用字段:智能] OR "预后营养指数"[常用字段:智能] OR "肠内营养"[常用字段:智能] OR "鼻饲"[常用字段:智能] OR "管饲"[常用字段:智能] OR "十二指肠管"[常用字段:智能] OR "胃残余量"[常用字段:智能] OR "胃潴留"[常用字段:智能] OR "喂养不耐受"[常用字段:智能] OR "身体成分"[常用字段:智能]) | 966629 |
| 5 | 1 AND 2 AND 3 AND 4 | 26 |
