# Supplementary material for: Point-of-care ultrasound in nutrition assessment and enteral nutrition management of critically ill children: a scoping review
Source: Front Nutr. 2026 Jun 22;13:1849240. doi: 10.3389/fnut.2026.1849240 (PMC13333426; doi:10.3389/fnut.2026.1849240)
Supplement: Supplementary file 2 [file Table_2.DOC]

Appendix B Methodological quality appraisal of included studies

| Study | Country | Study Design | Appraisal Tool | Key appraisal findings |
| --- | --- | --- | --- | --- |
| Farias (2025)[42] | Brazil | Review | JBI checklist for textual evidence: narrative | 6 yes. |
| Ichiro (2021)[23] | Japan | Retrospective cohort study | JBI checklist for cohort studies | 7 yes, 1 no, 3 N/A.  Limitations included single-center design and lack of confounder adjustment |
| Robert (2021)[36] | Boston | Prospective cohort study | JBI checklist for cohort studies | 10 yes, 1 no.  Limitations included single-center design and lack of confounder adjustment |
| Delia (2023)[50] | Spain | Prospective cohort study | JBI checklist for cohort studies | 10 yes, 1 no.  Limitations included single-center design and lack of confounder adjustment |
| Frederic (2017)[47] | France | Prospective cohort study | JBI checklist for cohort studies | 10 yes, 1 unclear.  Limitations included uncertainty regarding adjustment for confounders. |
| Alonso (2025)[37] | America | Diagnostic test accuracy study | JBI checklist for diagnostic test accuracy studies | 8 yes, 1 no, 1 N/A.  Limitations included small sample and discontinuous recruitment. |
| Ong (2017) [26] | Singapore | Review | N/A | N/A |
| Ruane (2021)[43] | Brazil | Prospective cohort study | JBI checklist for cohort studies | 10 yes, 1 unclear.  Limitations included single-center design and uncertainty regarding adjustment for confounders. |
| Jessica (2023)[41] | Brazil | Cross-section study | JBI checklist for analytical cross-sectional studies | 6 yes, 1 unclear, 1 no.  Limitations included small sample and uncertainty regarding adjustment for confounders. |
| Kay (2019)[35] | America | Cross-section study | JBI checklist for analytical cross-sectional studies | 7 yes, 1 no.  Limitations included single-center design and lack of confounder adjustment. |
| Jessica (2024)[51] | Brazil | Prospective cohort study | JBI checklist for cohort studies | 10 yes, 1 no.  Limitations included single-center design and lack of confounder adjustment. |
| Jinjiu (2025)[25] | China | Prospective cohort study | JBI checklist for cohort studies | 11 yes.  Limitations included single-center design. |
| Frederic (2022)[48] | France | Prospective cohort study | JBI checklist for cohort studies | 10 yes, 1 no.  Limitations included single-center design and lack of confounder adjustment. |
| Shereen (2025)[52] | Egypt | Prospective cohort study | JBI checklist for cohort studies | 11 yes.  Limitations included single-center design. |
| Ryan (2018)[10] | America | Prospective cohort study | JBI checklist for cohort studies | 11 yes.  Limitations included single-center design. |
| Agam (2022)[53] | India | Prospective cohort study | JBI checklist for cohort studies | 9 yes, 2 no.  Limitations included lack of confounder adjustment and failure to address loss to follow‑up. |
| Yunus (2016)[57] | Turkey | Diagnostic test accuracy study | JBI checklist for diagnostic test accuracy studies | 8 yes, 1 unclear, 1 N/A.  Limitations included small sample and single-center design. |
| Persson (2022)[19] | America | Review | JBI checklist for textual evidence: narrative | 6 yes. |
| Zamberlan (2023)[44] | Brazil | Review | JBI checklist for textual evidence: narrative | 6 yes. |
| Mohammed (2025)[38] | America | Prospective cohort study | JBI checklist for cohort studies | 11 yes.  Limitations included single-center design. |
| Lyvonne (2024)[54] | UK | Prospective cohort study | JBI checklist for cohort studies | 9 yes, 2 no.  Limitations included lack of confounder adjustment and failure to address loss to follow‑up. |
| Esther (2024)[39] | America | Prospective cohort study | JBI checklist for cohort studies | 9 yes, 2 no.  Limitations included lack of confounder adjustment and failure to address loss to follow‑up. |
| Tom (2015)[58] | Belgium | Cross-section study | JBI checklist for analytical cross-sectional studies | 7 yes, 1 no.  Limitations included single-center design and lack of confounder adjustment. |
| Yuan (2013)[45] | China | Quasi-experimental research | JBI checklist for quasi-experimental studies | 9 yes.  Limitations included single-center design. |
| Zhi (2025)[46] | China | Prospective cohort study | JBI checklist for cohort studies | 8 yes, 3 no.  Limitations included unadjusted confounders and unexplained loss to follow-up. |
| Claiborne (2021)[40] | America | Diagnostic test accuracy study | JBI checklist for diagnostic test accuracy studies | 8 yes, 1 no, 1 N/A.  Limitations included small sample and discontinuous recruitment. |
| Chengsi (2021)[55] | Singapore | Prospective cohort study | JBI checklist for cohort studies | 10 yes, 1 no.  Limitations included single-center design and unaddressed loss to follow-up. |
| Desgranges (2017)[49] | France | Prospective cohort study | JBI checklist for cohort studies | 10 yes, 1 no.  Limitations included single-center design and unadjusted confounders. |
| Adam (2015)[59] | Canada | Diagnostic test accuracy study | JBI checklist for diagnostic test accuracy studies | 10 yes.  Limitations included single-center design. |
| Edward (2020)[56] | Canda | Diagnostic test accuracy study | JBI checklist for diagnostic test accuracy studies | 9 yes, 1 N/A.  Limitations included single-center design. |
| Chikako (2016)[60] | Japan | Diagnostic test accuracy study | JBI checklist for diagnostic test accuracy studies | 5 yes, 4 no, 1 N/A.  Limitations included convenience sampling and unblinded design. |
| Flores (2006)[61] | Spain | Case report | JBI checklist for case reports | 8 yes. |
